# The endocannabinoid system as a therapeutic target in intestinal fibrosis

**DOI:** 10.3389/fphar.2025.1669951

**Published:** 2025-10-03

**Authors:** Zofia Misztal, Alicja Kaśniewska-Kosińska, Maria Wołyniak, Ewa Małecka-Wojciesko, Adam Fabisiak

**Affiliations:** Department of Digestive Tract Diseases, Faculty of Medicine, Medical University of Lodz, Lodz, Poland

**Keywords:** cannabinoid, inflammatory bowel disease, endocannabinoid system, fibrosis, inflammation, ulcerative colitis, Crohn’s disease

## Abstract

Intestinal fibrosis is a common and serious complication of inflammatory bowel diseases, often leading to strictures that require endoscopic or surgical intervention. Despite advances in anti-inflammatory therapies, effective antifibrotic treatments is currently not available. Therefore, new treatment methods for intestinal fibrosis are sought with the endocannabinoid system (ECS) as a potential therapeutic target. Cannabinoid receptors 1 and 2 (CB1/2) are classic receptors of the ES involved in the modulation of intestinal inflammation and permeability of the mucosal barrier. Experimental evidence from liver and lung models suggests that CB1 receptor activation promotes fibrosis through enhancement of the TGF-β/Smad pathway, interaction with the renin-angiotensin system, and upregulation of profibrotic markers, such as collagen and α-SMA. In contrast, CB2 receptor signaling appears to exert protective effects by limiting inflammation, fibroblast activation, and extracellular matrix deposition. Recent findings also suggest cross-talk between cannabinoid signaling and platelet-derived growth factor pathways, which are key drivers of myofibroblast proliferation and fibrogenesis. Although these mechanisms are well-established in hepatic, pulmonary and skin fibrosis, data from small and large intestine is scarce. However, direct evidence in intestinal fibrosis is scarce, representing a major knowledge gap. Elucidating ECS mechanisms in the alimentary tract could enable targeted antifibrotic strategies, complement current therapies, and reduce progression to fibrostenotic disease.

## 1 Introduction

Inflammatory bowel disease (IBD) is a group of diseases characterized by chronic inflammation of the gastrointestinal (GI) tract, with periods of exacerbation and remission. It consists of two main distinct pathologies: ulcerative colitis (UC), and Crohn’s disease (CD). In recent years, the prevalence of IBD has increased worldwide, with the highest incidence and pathogenicity observed in Northern Europe and North America (Ng et al., 2017). The course of IBD is often severe and aggressive, leading to serious complications, such as strictures or fistulas (Ungaro et al., 2016). These complications are mainly caused by the chronic, recurrent, and unresolved inflammatory processes, accompanied with intestinal fibrosis (Ungaro et al., 2016; Ananthakrishnan, 2021). Depending on the disease type, intestinal fibrosis may involve the mucosal and submucosal layers (UC), or the full width of the intestinal wall (CD). The clinical incidence of fibrosis is seen in more than 30% of CD patients and about 5% of UC patients (Latella et al., 2015). The mechanisms of intestinal fibrosis are complex and include both inflammation-dependent as well as independent factors. The progression of fibrosis is mainly driven by mesenchymal cells of the intestine (fibroblasts, myofibroblasts, and smooth muscle cells), which are contributing to the extracellular matrix (ECM) and crosslinking enzymes production. Those enzymes such as lysyl oxidases (LOX) and transglutaminases (TGase), mediate collagen crosslinking which influences the properties and structure of ECM (Latella et al., 2015; Kong et al., 2021). ECM accumulation leads directly to the tissue remodelling, fibrosis and narrowing of the intestinal lumen (Li et al., 2019). As a result of prolonged inflammation in IBD, tissue fibroblasts are activated and transformed into myofibroblasts, capable of producing ECM, and smooth muscle α-actin (α- SMA) is the known myofibroblast formation marker in GI tract. Myofibroblasts can differentiate into smooth muscle cells and cause the thickening of the muscularis propria, leading to the strictures formation.

**FIGURE 1:**
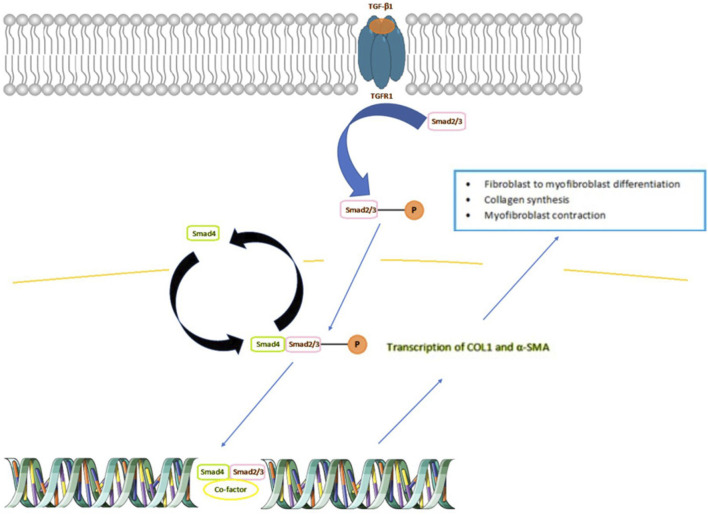
TGF-β/Smad pathway and its role in fibrogenesis. TGF-β, binds to a specific receptor on the plasma membrane: TGFR1, which enables phosphorylation and activation of Smad 2/3 proteins. The activated Smad 2/3 then binds to Smad4, which allows passage to the nucleus and leads to the transcription of the COL1 and α- SMA genes. As a result, the cell is transformed into myofibroblast, which is responsible for the formation and synthesis of components of the ECM and wound contraction. Abbreviations: COL1, Collagen, type I; α- SMA, smooth muscle α-actin; Smad, mothers against decapentaplegic; TGF, transforming growth factor; TGFR, transforming growth factor receptor. Adapted from (Verjee et al., 2013). Created with Scidraw.io.

In IBD, an upregulation of cytokines is observed, including transforming growth factor β (TGF-β). A specific member of this family of anti-inflammatory cytokines, TGF-β1, causes stricture formation via activation of downstream of so called “small mothers” proteins against decapentaplegic (Smad) signaling, leading to the overexpression of pro-fibrotic genes (Hu et al., 2018; Wang et al., 2022). In the colonic mucosa of IBD patients with intestinal strictures, TGF-β1 promotes the synthesis of collagen and ECM myofibroblasts contraction (Santacroce et al., 2022). The broader depiction of the role of TGF-β1 in fibrosis is shown in Figure 1. TGF-β, binds to a specific receptor on the plasma membrane: TGFR1, which enables phosphorylation and activation of Smad 2/3 proteins. The activated Smad 2/3 then binds to Smad4, which allows passage to the nucleus and leads to the transcription of the COL1 and α- SMA genes. As a result, the cell is transformed into myofibroblast, which is responsible for the formation and synthesis of components of the ECM and wound contraction.

When the fibrosis process is advanced, the intestinal lumen narrows causing the mechanical obstruction with the distention above. When prolonged, it leads to gradual damage of the intestinal wall, absorption loss and increase of intestinal wall permeability, which allows the penetration of toxins and bacterial translocation (Chan et al., 2018). Depending on the degree of stenosis and the degree of passage reduction, we distinguish partial and complete obstruction. In both types the therapeutic approach is either endoscopic or surgical. Depending on the inflammatory changes extent, the stenoses may occur in different localizations and therefore several segments resection is often required. Although the surgery rates in CD patients have decreased over the last few decades, the risk remain high with 46.6% after 10 years of diagnosis (Frolkis et al., 2013). Moreover, up to 24.2% of CD patients may require second surgery within the 5 years of the primary surgery (Frolkis et al., 2014). Additionally, severe, recurrent intestinal obstruction may lead to major resections and malnutrition or even the short bowel syndrome (Chan et al., 2018). Thus, each case the management type needs to be individually and carefully chosen, in order to limit the resected area.

Currently, the treatment of IBD focuses mainly on the use of anti-inflammatory drugs, such as aminosalicylates, glucocorticosteroids, immunosuppressants and biological therapy. New methods of disease management are sought which focus on inhibition or elimination of fibrosis (D’Haens et al., 2022; Butter et al., 2018). The proposed therapeutic targets for fibrosis in CD include: transforming growth factor (TGF)-β pathways, epithelial-mesenchymal transition, tissue inhibitor of metalloproteinase/matrix metalloproteinase (MMP) balance and endogenous cannabinoid system (ECS) (Bettenworth and Rieder, 2014). The latter appears to be a particularly interesting therapeutic target, which may accelerate wound healing and inhibit intestinal fibrosis. In this review we demonstrated the mechanisms behind the antifibrotic properties of ECS in intestinal inflammation.

## 2 The endocannabinoid system

The first ECS description contributed to the initiation of in-depth research on the regulation of the activity of this system in many human diseases, including multiple sclerosis, epilepsy, Alzheimer’s disease, and IBD (Mechoulam et al., 2014). ECS is a system consisting of “classical” and non-classical receptors, endogenous ligands (endocannabinoids) and enzymes that regulate their metabolism. The location of receptors translates into the role of the ECS, which is involved in the regulation of the following processes: sleep, mood, sensation, pain, appetite, digestion, memory, fertility and sex drive, immunity, muscle tension, inflammatory and metabolic processes in the body (Lowe et al., 2021). Research is currently underway to develop new drugs for the treatment of pain, immunosuppression, fibrosis and appetite regulation that would rely on the regulation of the activity of receptors or enzymes in the ECS (Lowe et al., 2021).

The “classical” cannabinoid receptors are G protein-coupled receptors (GPCR) and include CB1 and CB2 receptors. CB1 receptors are located mainly in the nervous system, including the brain, cerebellum and spinal cord. In addition, they are also expressed in the digestive, reproductive and immune system, lungs, heart, kidneys, endothelium of blood vessels and smooth muscles to a lesser extent (Izzo and Sharkey, 2010). CB2 receptors are present mainly in the peripheral nervous system, the immune and hematopoietic systems and the GI tract. With regard to the intestines, research on the ECS shows that CB1 and CB2 receptors are present in the entire enteric nervous system (ENS), which was confirmed in immunohistochemistry (IHC) (Duncan et al., 2005). The studies examined the expression of CB1 and CB2 receptors in the ENS in a guinea pig, rat and pig model. The ENS consists of the myenteric and submucosal plexuses, which are made of motor, sensory and interneurons. CB1 receptors are mainly located in excitatory motor neurons, interneurons, and primary afferent neurons. Both cannabinoid receptors are found in cholinergic neurons, colon crypt epithelial cells and lamina propria mononuclear cells (Izzo and Sharkey, 2010), (Duncan et al., 2005).

Endocannabinoids are the molecules, that influence the activity of cannabinoid receptors. The best-known endocannabinoids are arachidonoylethanolamine (anandamide, AEA) and 2-arachidonoylglycerol (2-AG). Anandamide is a partial agonist of CB1 and CB2, with a higher affinity to CB2, while 2-AG binds with comparable potency to both receptors and is present in greater amounts in the GI tract (Uranga et al., 2018). Released endocannabinoids bind to the cannabinoid receptors in the cell membrane, induce a biological response, and then are captured and inactivated by hydrolysis. Cannabinoid ligands primarily activate intracellular signaling pathways via Gi/o proteins, resulting in the suppression of adenylate cyclase activity and a subsequent reduction in cyclic AMP levels. Additionally, cannabinoids influence other intracellular targets, including the activation of p42 and p44 mitogen-activated protein kinases and the regulation of intracellular Ca^2+^ levels. Furthermore, the CB1 receptor is associated with various ion channels, such as N-type, P/Q-type, L-type calcium channels, and potassium channels (Demuth and Molleman, 2006). Anandamide is intracellularly hydrolyzed by N-acylethanolamine-hydrolyzing acid amidase and fatty acid amide hydrolase (FAAH), and 2-AG is hydrolyzed intracellularly by monoacylglycerol lipase (MAGL) and α/β-hydrolase domain 6 (Uranga et al., 2018).

In addition to the classical cannabinoid receptors, there are also non-classical cannabinoid receptors. They comprise a large group of receptors of non-cannabinoid origin, but also activated by cannabinoid ligands i.a. peroxisome proliferator-activated receptors (PPARs), transient receptor potential (TRP), free fatty acid receptors and other GPCRs. The modulation of these receptors activity was found to be implicated in diverse GI disorders. For instance, PPARs are nuclear hormone receptors consisting of three isoforms, α, δ and γ (O'Sullivan, 2016). PPARα is expressed in enterocytes, intestinal neurons of the myenteric and submucosal plexuses, as well as on glial cells (Sałaga et al., 2017). Although the effects of PPARα activation by cannabinoids in GI tract are not fully elaborated, yet evidence of their numerous functions in the intestines have been reported. In a study by De Filippos et al., PPARγ mediated the anti-inflammatory effect of cannabidiol (CBD) in lipopolysaccharide (LPS)-induced colitis in murine model. Those anti-inflammatory properties were shown with the suppression of mastocytes and macrophage infiltration and downregulation of TNF-α and chymase secretion (De Filippis et al., 2011). Apart from anti-inflammatory effect, an ongoing research suggests a PPARα activation influence the gut microbiota composition and intestinal permeability (Grabacka et al., 2022).

## 3 Endocannabinoid system in IBD

All the elements of the ECS are widespread in the GI tract and there is evidence that their expression changes significantly in IBD. It has been shown, that epithelial barrier damage and inflammation increase the cannabinoids expression. It has been shown, that *in vivo* mucosal CB1 expression is significantly elevated in inflamed colonic mucosa biopsies from CD patients (p < 0.001) and UC patients (p < 0.05), compared to the uninflamed mucosa (Di Sabatino et al., 2011). A similar relationship was demonstrated by examining the expression of AEA and its synthetic and degrading enzymes in IBD. However, their level can be either elevated or lowered, depending on the severity and course of the disease. In the acute phase of colitis in trinitrobenzene-sulfonic acid (TNBS)-induced mouse model of intestinal inflammation, a decrease in the level of FAAH mRNA was observed in inflamed region of colon, while in the late phase of inflammation, the level of FAAH was increased. Reduction of FAAH concentration resulted in an increase in the level of AEA, which in turn alleviated colitis by activating CB receptors. Conversely, in longstanding colitis activation of CB receptors was reduced (Storr et al., 2008). Moreover, it was shown that 3-day treatment with FAAH inhibitor, URB597, significantly reduced macroscopic damage score, myeloperoxidase (MPO) activity and colonic adhesions in inflamed mice, compared to mice treated with saline in a model of colitis, induced by intrarectal administration of TNBS (Storr et al., 2008). Furthermore, the results of study in colitis induced by intrarectal administration of dinitrobenzene sulfonic acid (DNBS) showed, that the FAAH-deficient mice had lower degree of colon inflammation, compared with their wild-type littermates. Level of inflammation was assessed using macroscopic scoring and MPO assay (Massa et al., 2004). In contrast, blocking CB1 receptor with SR141716A worsened the colitis in FAAH-deficient mice. On the other hand, 2-AG concentration remains unchanged in IBD, possibly due to the accelerated synthesis and degradation of 2-AG by diacylglycerol lipase-α and MAGL in the colon mucosa of UC patients (Marquéz et al., 2009). Regulation of cannabinoid receptors and activation by AEA and 2-AG have a positive effect on the healing of the epithelium and alleviating inflammation, not only in the parts of the intestine directly exposed to inflammatory factors, but systemically (Storr et al., 2008). Numerous *in vitro* and *in vivo* studies indicate that in various inflammatory conditions, cannabinoid receptors agonists downregulate mast cells and granulocytes and reduce cytokine release mainly affecting the CB2 receptors. In TNBS- and DSS-induced models of colitis, activation of CB2 receptor has been shown to contribute to the reduction of inflammatory cell influx, cytokine and chemokine secretion, and to improve macroscopic and histological inflammatory changes in the collected material. A study conducted in a mouse TNBS-induced model of colitis with CB1-, CB2- and CB1+2 double knockout mice strains showed an increase in intestinal inflammation in these groups. All knockout strains had severe inflammation, disturbed crypt architecture, edema and increased inflammatory infiltration. Inflammatory intensity was also confirmed with increased mRNA expression of inflammatory mediators, TNF-α and interleukin (IL)-1β and worsening of macroscopic scores. The above-mentioned data demonstrate the potential of modulating the ECS in treating intestinal inflammation, including IBD (Engel et al., 2010).

## 4 The role of classical cannabinoid receptors in the process of fibrosis

### 4.1 CB1

Cannabinoid receptors were found to affect the process of fibrosis on several levels. The modulation of cannabinoid receptors has been proven efficient in decreasing TGF-β1 in variety of fibrosis models. Krzyżewska et al. found that CBD reduced the expression of TGF-β1, galectin-3, SMAD2, pSMAD2 in monocratoline-induced pulmonary hypertension in rats (Krzyżewska et al., 2023). Moreover, CBD decreased the right ventricular interstitial and perivascular area of fibrosis in rats administered with monocratoline, while no differences were found in naive mice treated with CBD.

In the mouse model of acute liver wound repair induced by intraperitoneal injection of carbo tetrachloride (CCl(4)), the use of the selective CB1 receptor antagonist SR141716A decreased the expression of profibrotic markers such as TGF- β1 and α-SMA, which was associated with a reduction in the accumulation of fibrotic cells and inhibition of the activity of hepatic myofibroblasts (Teixeira-Clerc et al., 2006). A similar result was obtained in a study with animal model of skin fibrosis. It showed that both genetic (CB1^−/−^ knock-out mice) and pharmacological (using a CB1 receptor antagonist-AM281) inactivation of the CB1 receptor protects against the skin fibrosis process induced by bleomycin (Marquart et al., 2010).

The blockade of CB1 reduces fibrosis through the inhibition of angiotensin II (ANG II) signaling pathways (Rozenfeld et al., 2011) (Wengrower et al., 2012). The interplay between the renin-angiotensin system (RAS) and the ECS was explored in a study by Rozenfeld et al. (2011), which examined the mechanism of signal integration between the ANG II type 1 receptor (AT1R) and CB1 and their impact on ANG II-induced profibrogenic activity. The authors conducted an *in vitro* study, using activated hepatic stellate cells (HSCs) derived from ethanol-treated rats, in which CB1 was upregulated.

Importantly, a functional interaction between AT1R and CB1 has been identified, leading to the formation of AT1R–CB1 heteromers. Upregulation of these heteromers was shown to amplify ANG II-mediated signalling. Notably, stimulation of neuroblastoma cells with ANG II with the CB1 antagonist SR141716 inhibited ANG II-induced mitogenic signaling, whereas co-treatment with the CB1 agonist HU210 enhanced this response (Rozenfeld et al., 2011). Also, treatment with SR141716 blocked the overexpression of profibrogenic genes (α-SMA, TGF-β, pro-collagen αI and III) triggered by ANG II in HSCs from ethanol-treated rats.

Stellate cells are also present in the intestines, where they are referred to as intestinal subepithelial myofibroblasts (ISEMF) (Reynaert et al., 2003). ISEMF are found in the lamina propria, beneath the epithelial cells, and are considered an important factor in intestinal fibrosis (Rieder et al., 2017; Pucilowska et al., 2000). Increased CB1 expression in activated stellate cell, along with heteromerization with AT1R may enhance AT1R-mediated profibrotic activity, leading to excessive production of ECM components, including collagens.

Therefore, targeting CB1 receptor activity to modulate the molecular mediators of the RAS may hold therapeutic potential in preventing or attenuating intestinal fibrosis in IBD.

In a study by Di Sabatino et al. (2011), the effect of methanandamide (MAEA), a non-hydroxylable AEA analogue, on collagen production and migration of myofibroblasts was investigated. The wound-healing scratch assay was used to assess the impact of MAEA on wound healing. It was proven, that the concentration of soluble collagen was significantly lower in the culture of myofibroblasts isolated from intestinal strictures incubated with the 0.1 μM MAEA than in the culture without MAEA (140 ± 33 g/mL vs. 319 ± 72 g/mL, p < 0.01). The study showed that the MAEA significantly (p < 0.05) increased myofibroblast migration and accelerated wound healing (Di Sabatino et al., 2011).

The effect of another anandamide analogue, 2-methyl-2′-F-anandamide (Met-F-AEA), through the activity of 3-hydroxy-3-methylglutaryl coenzyme A (HMG-CoA) reductase on the fibrosis process was evaluated by Laezza et al. (2010). Activation of CB1 receptor by Met-F-AEA led to inhibition of HMG-CoA reductase through transcriptional regulation in human breast cancer cell lines, MDA-MB-231 and MCF7 (Laezza et al., 2010).

HMG-CoA reductase is a key enzyme responsible for catalyzing the conversion of HMG-CoA to mevalonic acid, which is a substrate in the synthesis of endogenous cholesterol and formation of isoprenoid intermediates. Isoprenoids, in turn, are involved in the activation of the Rho/ROCK pathway, which is responsible for the development of fibrosis, inflammation, and vascular damage. The role of the Rho/ROCK pathway in intestinal fibrosis is to control the expression of connective tissue growth factor (CTGF), which mediates myofibroblast differentiation, fibroblast proliferation and collagen synthesis (Gervaz et al., 2009). Thus, modulation of CB1 activity indirectly inhibits the fibrosis process by decreasing the isoprenoids level and Rho/ROCK pathway by lowering the HMC-CoA reductase activity.

Although promising outcomes have been achieved in preclinical studies in alleviating fibrosis intensity in various tissues and models, the complexity of the ECS defines its limitations. Interesting results were provided by Aljobaily et al. in a study concerning the utility of cannabigerol (non-psychoactive cannabinoid) in treating non-alcoholic steatohepatitis (NASH) (Aljobaily et al., 2022). Briefly, mice were fed with diet deficient in methionine/choline or control diet and after 3 weeks 3 subgroups were distinguished: mice treated with low or high dose of cannabigerol, or vehicle. In low-dose group mice livers exhibited lower and in high-dose -increased collagen deposition, both compared to control mice. The sparsity of results may derive from the specific choice of ligand as cannabigerol is a weak partial agonist of CB1 and CB2, but also weak agonist of TRPV1-3, TRPA1, PPAR-γ, highly potent agonist of α2-adrenergic receptor and moderately potent antagonist of serotonin 5-HT1A receptor (Calapai et al., 2022). Also, in another study, CBD a reduced the hepatic steatosis in another murine model of NASH (high-fat high cholesterol diet) (Huang et al., 2019). The effect was confirmed by significant reduction in alanine aminotransferase, serum total cholesterol and triglicerides in treated mice compared to mice without the treatment. Moreover, IHC staining revealed lower CD68^+^ macrophages in the liver of treated mice, while RT-PCR showed significanly decreased IL‐1β, TNF‐α, and monocyte chemoattractant protein-1 (MCP-1). Cannebigerol may have failed to achieve this in the aforementioned study due to lower specificity (Huang et al., 2019). The use of more selective cannabinoid ligands appears crucial to avoid such discrepancies in further studies.

### 4.2 CB2

**FIGURE 2:**
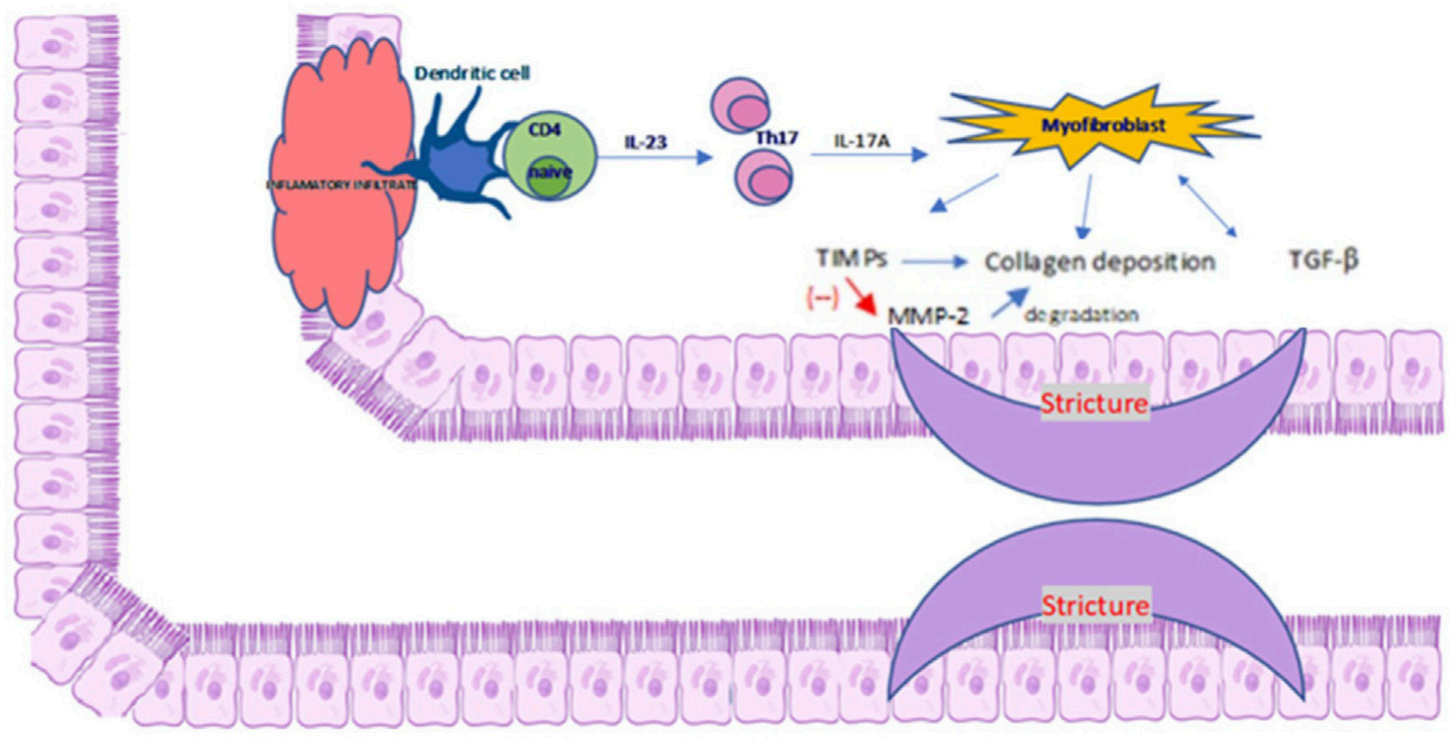
Cellular and molecular mechanisms of inflammatory infiltration involved in the formation of stricture in IBD. Intestinal mucosa infiltration of CD4^+^ cells stimulate Th17 cells to produce profibrotic cytokines, in particular IL-17A, which inducts the collagen and TIMP secretion from intestinal myofibroblasts and inhibition of their migration. TIMP inhibits MMP-2 production, contributing to ECM proteins degradation. This leads to an increased collagen deposition. Those processes contribute to the formation of intestinal strictures. Abbreviations: ECM, extracellular matrix; CD4, cluster of differentiation 4; IL-17A, interleukin-17A; MMP-2, matrix metalloproteinase 2; TIMP, tissue inhibitor of matrix metalloproteinase; Th17, T helper 17 cells. Adapted from (Santacroce et al., 2022). Created with Scidraw.io.

The activation of CB2 evokes antifibrotic properties as demonstrated in a rat model of liver cirrhosis and ascites. The administration of the selective agonist 3-(1,1-dimethylbutyl)-1-deoxy-∆8-tetrahydrocannabinol (JWH-133) to rats for 9 days reduced inflammatory infiltration, α- SMA expression, type I collagen storage and increased the MMP-2 expression (Muñoz-Luque et al., 2008). MMP-2 is involved in the degradation of ECM proteins, which inhibits abnormal collagen deposition. A key role in fibrosis plays inflammatory infiltration of T helper (Th) cells, especially Th1, Th2, Th17. Th1 cells secrete interferon (INF)-γ and IL-12, Th2 and Th17 secrete interleukin 17 and 22. In particular, IL-17A stimulates myofibroblasts to secrete collagen and tissue inhibitor of MMP and prevents myofibroblast migration. A detailed description of the relationship between the cellular and molecular mechanisms of inflammatory infiltration in fibrosis is presented in Figure 2. Similar results were obtained by examining the effect of CB2 receptor activation with the use of a specific agonist (GP1a) in the mouse model of skin wound healing. It has been shown that GP1a evoked a reduction in skin fibrosis through a decrease in collagen deposition and decrease in the level of TGF-β1 and Smad3 following post-traumatic skin damage in mice (Li et al., 2016; Correia-Sá et al., 2020). In addition, genetic inactivation of the CB2 receptor or pharmacological inactivation with the use of specific antagonist (AM630) abolished the protective antifibrotic effect of CB2. In studied mice, the wounded group administered with AM630 exhibited increased deposition of collagen and thick fibers, compared with to the control group (Correia-Sá et al., 2020).

The role of CB2 receptor in fibrosis was also evaluated in a mouse model of liver fibrosis (LF) (He et al., 2017). Liver fibrosis model was induced by intraperitoneal injections of 30% carbon tetrachloride CCl(4). Next, mice were divided into four groups: control group, model group with induced LF and two groups with induced LF treated with CB2 agonist, AM1241. In case of the group with induced LF, significantly increased level of platelet-derived growth factor (PDGF) and collagen type III (Col-III) were observed compared to the control group (p < 0.05). Treatment with AM1241 at any dose reduced the level of fibrosis and significantly reduced expression of PDGF and Col-III compared to the model group (p < 0.05) (He et al., 2017). PDGF is a potent mitogen with properties similar to TGF-β. Its secretion is significantly increased in chronic inflammation. After binding to the tyrosine kinase PDGF receptor (PDGFR), it affects the chemotaxis and proliferation of fibroblasts (Gervaz et al., 2009). There are five isoforms of PDGF: AA, -AB, -BB, -CC, -DD, which combine with the appropriate PDGFR dimers: αα, αβ or ββ. Among isoforms of PDGF, the most important are PDGF-C which is secreted in the stomach in the smooth muscle layer, small and large intestine and PDGF-B secreted in colonic mucosa. These molecules by binding to selected PDGFR like PDGFR-β stimulate intestinal myofibroblasts to migrate, proliferate and produce collagen (linkhammer et al., 2018). Studies conducted in material from CD patients, as well as in plasma and serum of IBD patients, showed significantly elevated levels of PDGF-β (Fan et al., 2014). Studies in animal models of lung and liver fibrosis have demonstrated that PDGFR-β/PI3K/Akt signalling pathway is involved in proliferation, transformation of HSC/myofibroblasts and deposition of collagen (Fan et al., 2014).

Notably, Liu et al. also indirectly suggested an interplay between cannabinoid and PDGF signalling (Liu and Shi, 2019). The authors demonstrated that pirfenidone, a medication approved for the treatment of idiopathic pulmonary fibrosis (IPF), exerts anti-inflammatory and antifibrotic effects through activation of the CB2 receptor in a bleomycin-induced mouse model of IPF. When human embryonic lung fibroblasts (WI38) were incubated with bronchoalveolar lavage fluid from IPF mice, pirfenidone reduced fibroblast activity. However, co-treatment with pirfenidone and the CB2 antagonist SR144528 abolished the anti-inflammatory and anti-fibrotic effects, indicating CB2-dependency. Moreover, this combination reversed the suppression of fibroblast activity, a process in which PDGF signalling is known to play a key role, suggesting a potential functional interaction between CB2 and PDGF pathway.

In view of the presence of PDGFR-α, expressed in the intestinal mesenchymal cells, and their proven participation in the fibrosis process, it seems that they may also play a significant role in fibrosis and stricture formation in the course of IBD. On the other hand, the CB2 agonist, reducing the expression of PDGFR in the intestines, could become one of the therapeutic targets.

Literature provides evidence that one of possible substances affecting classical cannabinoid receptors, which has also antifibrotic properties, may be CBD. In a rat model of endometriosis oral administration of CBD fwas associated with reduction in collagen deposition in endometriotic lesions in Masson trichrome staining, compared to vehicle-treated rats (Genovese et al., 2022). Moreover, a significant decrease in the expression of matrix metalloproteinase-9 (MMP-9), inducible nitric oxide synthase (iNOS) and TGF-β was noted (Genovese et al., 2022). MMP-9 is involved in the wound healing process, ECM degradation, and angiogenesis. It belongs to the endopeptidases contributing to the degradation of collagen, gelatin and the basement membrane. In addition, it regulates cell migration, signalling and invasion, and epithelial-to-mesenchymal transition (Goffin et al., 2016).

The effect of selective anti-MMP-9 monoclonal antibodies on fibrosis was investigated in a mouse model of intestinal fibrosis after heterotropic intestinal transplantation. Anti-MMP-9 antibodies, CALY-001, AB-0046-h4 and isotype control antibody was administered to mice intraperitoneally. Quantitative analysis of the collected samples showed that the collagen layer in the grafts from mice receiving anti-MMP-9 antibodies is significantly thinner, compared to the sample treated with the isotype control (p < 0.0001). An additional confirmation of those was the low level of the collagen-specific amino acid, hydroxyproline, in the samples treated with anti-MMP-9 antibodies, compared to the control ones (Goffin et al., 2016).

iNOS is an enzyme responsible for the synthesis of nitric oxide (NO) in macrophages, involved in the body’s defence reactions. In case of chronic inflammation, excessive production of NO may occur, leading to oxidative stress. When prolonged, it damages cellular components and may lead to fibrosis. The exact mechanism by which iNOS influences fibrosis is not fully understood, but the induction of hypoxia-inducible factor 1 α (HIF-1α),MMP-9 and the promotion of DNA damage by iNOS are being considered (Anavi et al., 2015). The role of iNOS in the process of fibrosis was investigated in a cholesterol-induced liver fibrosis mouse model by feeding wild-type and iNOS-deficient mice on a high cholesterol diet (HCD) or a control diet for 6 days. Western blot analysis showed significantly increased expression of collagen type I and profibrotic cytokines TNFα and TGF-β in HCD-fed wild-type compared to HCD-fed iNOS-deficient mice. In addition, iNOS has been shown to increase HIF-1α in wild type HCD, which was accompanied by an increase in the expression of profibrotic peptides, such as PDGF-A, PDGF-B. The lower level of MMP-9 was noted in HCD-fed iNOS-deficient mice than in wild type (Anavi et al., 2015). Taking all this into account, it can be concluded that the CBD, MMP-9 and iNOS expression reduction, may have the anti-fibrotic effect.

Moreover, CBD has been shown to influence activated HSCs by promoting their apoptosis and thus regression of fibrosis. Such conclusions were led by a study carried out in HSC line derived from cirrhotic patient, as well as rat and mouse HSCs lines activated with ethanol or CCl4 (Lim et al., 2011). Isolated and cultured activated HSCs were exposed to CBD at various concentrations for 2, 4 and 8 h. Then cell viability was measured using the acid phosphatase assay, cell death was measured using Western blot analysis and the CBD-induced apoptosis was assessed with flow cytometric analysis of FITC-Annexin V staining. Activated HSCs are an important element in liver fibrosis, as they stimulate ECM proteins secretion. The apoptotic effect was characteristic for activated HSCs causing the fibrosis in contrast to normal hepatocytes (Lim et al., 2011).

Table 1 summarizes the mechanisms underlying CB1/2 modulation in fibrosis.

**TABLE 1:** Antifibrotic effects of cannabinoid receptor modulation.

Receptor	Receptor activity	Cumulative anti-fibrotic effect
CB1 receptor	Receptor blockade	↓ Expression of profibrotic TGF-β/Smad pathway, α-SMA (Krzyżewska et al., 2023; Teixeira-Clerc et al., 2006) Inhibition of signalling pathways mediated by ANG II (Rozenfeld et al., 2011; Wengrower et al., 2012) • Rho cascades • MAP kinase • JAK/STAT Inhibition of transmission of mitogenic signals (Rozenfeld et al., 2011) Inhibition of profibrogenic genes (Rozenfeld et al., 2011) ↓ Expression of CTGF (Laezza et al., 2010)
CB2 receptor	Receptor Activation	↓ Collagen deposition (Muñoz-Luque et al., 2008; Li et al., 2016; Correia-Sá et al., 2020) ↓ Level of TGF-β1/Smad3 (Muñoz-Luque et al., 2008; Li et al., 2016; Correia-Sá et al., 2020) ↓ Expression of PDGF, Col-III (He et al., 2017; Liu and Shi, 2019)
CB1 and CB2 receptors	Receptors activation	↑ Migration of myofibroblasts accelerating wound healing (Di Sabatino et al., 2011) ↓ Activity of HMG-CoA reductase (Laezza et al., 2010) Inhibition of Rho-ROCK signaling pathway (Laezza et al., 2010) Inhibition (Di Sabatino et al., 2011; Marquart et al., 2010; Gervaz et al., 2009; He et al., 2017) of • myofibroblast differentiation • fibroblast proliferation • collagen synthesis ↓ MMP-9, iNOS, TGF-β expression (Genovese et al., 2022; Goffin et al., 2016) Exacerbation of existing ER stress in activated stellate cells (Lim et al., 2011) Apoptosis of activated stellate cells producing ECM (Lim et al., 2011)

The table presents the cumulative antifibrotic actions resulting from either blockade of the CB1 or activation of the CB2, as well as combined activation of both receptors. Mechanisms include modulation of profibrotic signaling pathways (e.g., TGF-β/Smad, ANG II-mediated cascades), inhibition of profibrogenic gene expression, reduction of collagen deposition, and induction of apoptosis in activated stellate cells. Reported downstream effects involve decreased myofibroblast differentiation, fibroblast proliferation, collagen synthesis, and extracellular matrix ECM, production. Abbreviations: TGF-β, transforming growth factor β; ANG II, angiotensin II; CTGF, connective tissue growth factor; PDGF, platelet-derived growth factor; Col-III, collagen type III; MMP-9, matrix metalloproteinase-9; iNOS, inducible nitric oxide synthase; ER, endoplasmic reticulum; ECM, extracellular matrix.

## 5 The role of non-classical cannabinoid receptors in the process of fibrosis

TRP ion channels, including the TRPV and TRPA subfamilies, have a significant role in regulation of inflammation and pain in IBD. Inflammatory factors, prostaglandins, bradykinin, and proteases upregulate these receptors in UC and CD patients (Csekő et al., 2019). Increased expression of myofibroblast TRPA1 channels has been noted in colonic strictures in CD patients (Hiraishi et al., 2018). In addition to its anti-inflammatory properties, TRPA1 activation has been shown to protect against intestinal fibrosis, by the inhibition of TGF-β1 and α- SMA-dependent collagen production (Hiraishi et al., 2018). Moreover, human intestinal myofibroblast cell culture study showedthat TRPA1 expression increased after the addition of type I collagen to the medium. As a result, endogenous production of collagen was decreased. The result suggests that the activity of the channel negatively corelates with collagen synthesis in intestinal myofibroblasts, indicating antifibrotic effect worth exploring (Hiraishi et al., 2018).

PPAR-γ agonists have been shown to reduce fibrosis in many organs, including the intestines, while selective PPAR-γ antagonists abolish these effects (Di Gregorio et al., 2017). It was showed that the PPAR-γ receptor modulator, GED- 0507-34 Levo (GED), reduced the expression of fibrotic markers, such as α-SMA, collagen I-III, fibronectin and pro-fibrotic molecules (IL-13, TGF- β and Smad3) in a mouse model of 2.5% DSS-induced intestinal fibrosis. The significant decrease in the expression of the fibrotic factors (α-SMA, collagen I-III and fibronectin) at protein (p = 0.001, p = 0.015, p = 0.015, accordingly) and mRNA (p = 0.043, p = 0.0003, p = 0.040 accordingly) level was noted in mice treated with GED, compared to inflamed mice (Di Gregorio et al., 2017).

A similar result was obtained by examining the effect of PPAR-γ activation with ajulemic acid, the non-psychoactive synthetic analogue of THC in skin fibrosis (Correia-Sá et al., 2020; Gonzalez et al., 2012). Ajulemic acid reduced progression of fibrosis by reducing collagen deposit (p < 0.05) in bleomycin-induced dermal fibrosis, compared to untreated mouse (Gonzalez et al., 2012). A similar result was obtained by examining the effect of PPAR-γ activation with ajulemic acid, the non-psychoactive synthetic analogue of THC in skin fibrosis (Correia-Sá et al., 2020; Gonzalez et al., 2012). Ajulemic acid reduced progression of fibrosis by reducing collagen deposit (p < 0.05) in bleomycin-induced dermal fibrosis compared to untreated mouse (Gonzalez et al., 2012).

Table 2 summarizes the preclinical studies discussed in the manuscript.

**TABLE 2:** Summary of preclinical studies on cannabinoid modulation in fibrosis.

Study	Model	Intervention	Effect
Krzyżewska et al. (2023)	Monocratoline-induced pulmonary hypertension in rats	CBD (10 mg/kg) OD for 21 days	Reduced in expression of TGF-β1, galectin-3, SMAD2
Aljobaily et al. (2022)	Mice fed with methionine/cholin-deficient diet	Low or high dose of CBG	Low-dose CBG reduced hepatic collagen deposition, whereas high-dose CBG increased deposition
Huang et al. (2019)	Murine model of NASH induced by a high-fat, high-cholesterol diet.	CBD (5 mg/kg) administered intragastrically OD for 8 weeks	Reduced hepatic steatosis
He et al. (2017)	Liver fibrosis induced by intraperitoneal injections of 30% carbon tetrachloride (CCl_4_), 3 times a week at 5 mL/kg for 16 weeks	CB2 agonist AM1241 (3 or 9 mg/kg) for 16 weeks	Reduced fibrosis and significantly decreased expression of PDGF and collagen type III.
Genovese et al. (2022)	Rat model of endometriosis induced by intraperitoneal injection of minced uterine tissue from donor rats pretreated with pregnant mare serum gonadotropin	CBD (10 mg/kg) orally for 7 consecutive days	Reduced collagen deposition and decreased expression of MMP-9, iNOS, and TGF-β in endometriotic lesions compared to vehicle-treated rats
Goffin et al. (2016)	Mouse model of intestinal fibrosis after heterotropic intestinal transplantation	From day 5 post-procedure, mice received intraperitoneal injections of anti-MMP-9 antibodies (CALY-001, AB-0046-h4) or isotype control antibody at 30 mg/kg every 3 days until day 11	Mice treated with anti-MMP-9 antibodies showed a significantly thinner collagen layer in grafts and reduced levels of the collagen-specific amino acid hydroxyproline compared with controls
Lim et al. (2011)	Human HSC line derived from a cirrhotic patient, rat and mouse HSC lines activated with ethanol or CCl4	Activated HSCs were treated with CBD at various concentrations for 2, 4, and 8 h. Cell viability was assessed by acid phosphatase assay, cell death by Western blot, and CBD-induced apoptosis by flow cytometric analysis using FITC-Annexin V staining	CBD induced apoptosis selectively in activated HSCs, which contribute to fibrosis, but not in normal hepatocytes
Di Gregorio et al. (2017)	Mouse model of 2.5% DSS-induced intestinal fibrosis	GED-0507-34 Levo was administered by oral gavage at 30 mg/kg/day starting on day 12, at the beginning of the second of three DSS cycles	GED- 0507-34 Levo, reduced the expression of fibrotic markers, (α-SMA, collagen I-III, fibronectin) and pro-fibrotic molecules (IL-13, TGF-β, and Smad3)

α-SMA, alpha smooth muscle actin; CBD, cannabidiol; CBG, cannabigerol; CCl4, carbon tetrachloride; Col-III, collagen type III; DSS, dextran sulfate sodium; HSC, hepatic stellate cell; IL, interleukin; iNOS, inducible nitric oxide synthase; MMP-9, matrix metalloproteinase-9, NASH, non-alcoholic steatohepatitis; PDGF, platelet-derived growth factor; TGF-β, transforming growth factor β.

## 6 Limitation and future directions

Research on the influence of ECS on organ fibrosis remains limited. The underlying mechanisms along with the therapeutic potential in hepatic, pulmonary, and dermal fibrosis have only began to be explored in recent years. However, the evidence for the role of cannabinoids in intestinal fibrosis remain scarce. Notably, intestinal fibrosis is influenced by complex factors (chronic inflammation, gut microbiota, and mechanical stress) limiting the direct extrapolation of findings from other organs. A deeper understanding of the fibrosis process in IBD, as well as the impact of ECS on the inflamed gut requires further investigation, as the demand for novel antifibrotic treatments for IBD patients remain critical.

Although classical cannabinoids have shown beneficial effects *in vitro* and *in vivo* models of intestinal fibrosis the influence on central nervous system is not negligible. Thus, the strategies targeting non-classical cannabinoid receptors or peripheral classical receptors are more safer therapeutic alternatives. Future advances will require well-designed preclinical studies using appropriate animal or organoid models, combined with non-invasive biomarkers of fibrosis and optimized administration strategies. Additionally, investigating the adaptation of existing antifibrotic therapies for intestinal strictures may provide complementary approaches. Nonetheless, the direct translation of antifibrotic therapies from other organs does not seem to be achievable in the near future (Bettenworth and Rieder, 2014).

## 7 Conclusion

The ECS is widespread in the human body, which proves its many functions in the body. Due to its presence in the digestive system and immune cells, it can influence the modulation of inflammation and the process of fibrosis in IBD. Numerous studies, both in animal models, cell cultures and in human tissue, show that the activation or inhibition of individual elements of the ECS can affect the process of intestinal fibrosis. Hence, the ECS may be a potential target aiming at the fibrosis reduction. Additional therapy with anti-fibrotic agents in subpopulation of patients with high stenosis risk, such as fibrotic phenotype CD patients, may prove useful in preventing the IBD complications. The most prominent and recurring mechanisms in research involving ECS include modulation of TGF-β/Smad pathway, RAS, direct inhibition of pro-fibrotic genes, and crosstalk with the PDGF signaling pathway. However, further preclinical studies are needed, as current knowledge is primarily derived from models of liver or skin fibrosis. Despite the high interest in anti-fibrotic therapy, no interventional clinical trials involving ECS are currently ongoing, according to ClinicalTrials.gov or Pubmed. This is most likely due to undesired effects connected to the treatment with classic cannabinoids. However, new strategies for treating inflammatory diseases of the GI tract, based on the activation of endocannabinoid receptors and the regulation of cannabinoids, arise as potential therapeutics in this indication.
